# Rice Husk, Brewer’s Spent Grain, and Vine Shoot Trimmings as Raw Materials for Sustainable Enzyme Production

**DOI:** 10.3390/ma17040935

**Published:** 2024-02-17

**Authors:** Ana Guimarães, Ana C. Mota, Ana S. Pereira, Ana M. Fernandes, Marlene Lopes, Isabel Belo

**Affiliations:** 1 Centre of Biological Engineering, University of Minho, 4710-057 Braga, Portugalmarlenelopes@ceb.uminho.pt (M.L.); 2 LABBELS—Associate Laboratory, 4710-057 Braga, Portugal; 3 CITEVE—Technological Centre for the Textile and Clothing Industry, 4760-034 Vila Nova de Famalicão, Portugal

**Keywords:** agro-industrial by-products, *Aspergillus niger*, lignocellulolytic enzymes, simplex centroid mixture design, solid-state fermentation

## Abstract

Solid by-products with lignocellulosic structures are considered appropriate substrates for solid-state fermentation (SSF) to produce enzymes with diverse industrial applications. In this work, brewer’s spent grain (BSG), rice husk (RH), and vine shoot trimmings (VSTs) were employed as substrates in SSF with *Aspergillus niger* CECT 2088 to produce cellulases, xylanases, and amylases. The addition of 2% (NH_4_)_2_SO_4_ and 1% K_2_HPO_4_ to by-products had a positive effect on enzyme production. Substrate particle size influenced enzyme activity and the overall highest activities were achieved at the largest particle size (10 mm) of BSG and RH and a size of 4 mm for VSTs. Optimal substrate composition was predicted using a simplex centroid mixture design. The highest activities were obtained using 100% BSG for *β*-glucosidase (363 U/g) and endo-1,4-*β*-glucanase (189 U/g), 87% BSG and 13% RH for xylanase (627 U/g), and 72% BSG and 28% RH for amylase (263 U/g). Besides the optimal values found, mixtures of BSG with RH or VSTs proved to be alternative substrates to BSG alone. These findings demonstrate that SSF bioprocessing of BSG individually or in mixtures with RH and VSTs is an efficient and sustainable strategy to produce enzymes of significant industrial interest within the circular economy guidelines.

## 1. Introduction

Recent previsions indicate that the world’s population will increase to 9.9 billion by 2050. Feeding a constantly growing population will require a significant increase in food production [1,2]. Consequently, the higher demand for food will intensify environmental pollution and will generate massive quantities of agro-industrial by-products. Globally, it is estimated that the food industry produces approximately 1.3 billion tons of by-products [3]. The processing of important food commodities such as cereals, vegetables, fruit, wine, and beer produces nonedible residues and wastes of a lignocellulosic nature (e.g., husks, straws, stalks, oil seed cakes, brewer’s spent grains, pomaces, pulps, leaves, barks, etc.) that are dismissed by the industry and usually rejected as waste [4]. Most of these residues are either disposed of in landfills, incinerated, composted, or, in some cases, used as low-value animal feed [5].

These by-products are a source of lignocellulosic biomass and are rich in bioactive molecules, enzymes, and fibers [3,6,7]. For decades, there have been concerted efforts to develop methodologies to convert these residues into valuable resources in a circular economy perspective, which encourages the reusability of by-products, reduces waste, and generates profit from discarded material [8,9,10]. Brewer’s spent grain, rice husk, and vine shoot trimmings are lignocellulosic by-products generated in large quantities worldwide. Brewer’s spent grain (BSG) is the main by-product of the brewing industry, representing around 85% of the total by-products generated [11]. BSG mostly comprises the husk–pericarp–seed coat layers that cover the barley grain and other residual solids formed after the wort formation stage. BSG is a lignocellulosic material that consists of 17% cellulose, 28% non-cellulosic polysaccharides, arabinoxylans, and lignin. Despite its potential due to its high content of protein and fiber, its main application is currently limited to animal feed [12]. Rice husk (RH) is the most abundant residue resulting from the processing of rice and represents approximately 20% to 25% of the total weight of the rice grain [13]. It is a cellulose-based fiber that contains 20% silica in amorphous form. Approximately 120 million tons of RH are generated every year, and disposal of this by-product is often accomplished by incineration [14]. The wine industry, one of the most socio-economically important sectors, generates around 20 million tons of by-products annually, including vine shoot trimmings (VSTs) [15]. Though wine by-products are rich in phenolic compounds and other biomolecules, around 40% of these by-products are still combusted, which contributes to increased pollution [16].

The processing of agro-industrial by-products by solid-state fermentation (SSF) to obtain economically valuable microbial products has been proposed as a suitable strategy for responding to the ecological and economic problems of the agri-food industry [17,18,19,20]. SSF is defined as the fermentation process of a solid substrate in the absence or near absence of water [21]. In general, the microorganisms best suited to grow in SSF are filamentous fungi since SSF substrates simulate their natural habitat. The hyphal mode of fungal growth that allows them to penetrate the spaces between substrate particles and their tolerance to lower water activities and high osmotic pressure are the main advantages in the colonization of solid substrates and the obtention of available nutrients [22]. Moreover, filamentous fungi are efficient producers of several biomolecules of interest such as enzymes, single-cell proteins, aromatics, pigments, phenolics, and other compounds [23,24,25,26,27].

SSF presents several advantages over other types of fermentations (e.g., submerged fermentation), as it allows the use of raw materials as a physical matrix for microorganisms while also providing nutrients that support microbial growth. Target molecules can be produced with high yields/productivity at low energy costs, using less water and a smaller fermentation area, and with easier downstream processing [22,28].

In the last decade, lignocellulolytic enzymes have been among the most studied biomolecules to be produced through the SSF of agro-industrial by-products [19,23,29]. These are formed in fermentations conducted specifically by fungi and are produced to facilitate the degradation of cell-wall polysaccharides, cellulose, and hemicellulose into fermentable sugars [30]. These enzymes have applications as biocatalysts in several processing industries such as the food, feed, paper, biofuel, textile, leather, and pharmaceutical industries.

This work aimed to evaluate the use of the agri-food by-products brewer’s spent grain, rice husk, and vine shoot trimmings in SSF to obtain value-added enzymes. In this study, the target enzymes were mainly cellulases and xylanases, but amylase was also assessed. Factors affecting enzyme production by SSF were studied such as solid particle size, nitrogen and phosphorus supplementation, and fermentation time. Moreover, mixtures between the three by-products were studied, using a simplex centroid mixture design in the experiments, to find the optimal substrate for enzyme production. Under the optimized conditions, the effect of fermentation time was also evaluated.

## 2. Materials and Methods

### 2.1. Microorganisms

*Aspergillus niger* CECT 2088 was obtained from the Coleción Española de Cultivos Tipo (CECT Valencia, Spain) and stored at −80 °C in an aqueous solution of 1% (*w*/*v*) peptone and 30% (*v*/*v*) glycerol. The fungal strain was revived in PDA medium (potato extract 4 g/L, dextrose 20 g/L, agar 15 g/L,) for 7 days at 25 °C.

### 2.2. Raw Materials

Brewer’s spent grain (BSG), rice husk (RH), and vine shoot trimmings (VSTs) were supplied by LETRA craft brewery (Vila Verde, Portugal), Nova Arroz company (Oliveira de Azeméis, Portugal), and small-scale winery (local variety Loureiro and Touriga Nacional) (Famalicão, Portugal), respectively. These materials were dried (<10% moisture) and ground to different particle sizes (1 mm, 4 mm, and 10 mm) using a Retsch SM 300 cutting mill (Retsch GmbH, Haan, Germany). All materials were stored in hermetic bags at room temperature.

### 2.3. Characterization of Solid Substrates

The agro-industrial by-products were physically and chemically characterized. Moisture was calculated by drying the substrates in an oven at 105 °C until constant weight. Ash content was determined by high-temperature exposure at 550 °C for 2 h in a muffle furnace (Nabertherm, Lilienthal, Germany). Total nitrogen content was determined by the Kjeldahl method, from which total protein content was obtained using a conversion factor of 6.25. The total lipid content was quantified by the Soxhlet method using petroleum ether as a solvent at 70 °C. The lignocellulosic composition (cellulose, hemicellulose, and Klason lignin) of the substrates was determined by quantitative acid hydrolysis in a two-stage acid treatment. The first stage consisted of hydrolysis at 30 °C for 1 h with 72% H_2_SO_4_, and the second stage was carried out after the dilution of hydrolysate with water at 121 °C for 1 h. The solution was filtered through a Gooch crucible to retain lignin, which was dried at 105 °C. The filtrate was analyzed using a high-performance liquid chromatography system using a Jasco830-IR intelligent refractive-index detector (Jasco, Tsukuba, Japan) and a Varian MetaCarb 87H column (Agilent, Santa Clara, CA, USA). The column was eluted with 0.005 M H_2_SO_4_, with a flow rate of 0.5 mL/min at 60 °C [31].

Mineral content (barium, calcium, copper, iron, potassium, magnesium, manganese, sodium, phosphorus, strontium, and zinc) was determined by ICP-OES (PerkinElmer, Inc., Waltham, MA, USA) after microwave-assisted digestion. Briefly, 10 mL HNO_3_ and 2 mL H_2_O_2_ were added to 500 mg of RH, 10 mL HNO_3_ was added to 600 mg of BSG, and 10 mL HNO_3_ and 3 mL HCl were added to 250 mg of VSTs. Samples were digested at 170 °C for 10 min at 30 bar, followed by digestion at 200 °C for 15 min for RH and BSG. For VSTs, samples were digested at 200 °C for 35 min at 35 bar. Samples were diluted to an acid concentration of approximately 2.5% (*v*/*v*) and filtered with 0.2 μm nylon filters. The operation conditions of analysis were the following: radio frequency power of 1300 W, argon plasma flow of 10 L/min, auxiliary gas flow of 0.2 L/min, and nebulizer gas flow of 0.7 L/min.

To estimate soluble protein, free sugars, and enzymatic activity a solid–liquid extraction with distilled water (ratio of 1 g of dry solid to 10 mL of water) was performed at 20 °C for 30 min under the agitation of 200 rpm. The liquid extract was obtained by filtering the mixture through a nylon mesh filter and centrifuging for 10 min at 4 °C and 7000× *g* rpm. The liquid extract was recovered and stored at −20 °C until further analysis. The soluble protein in the filtrate was quantified by the Bradford method [32], and free sugars were determined by the 3,5-dinitrosalicylic acid (DNS) method [33].

Table 1 shows the composition of BSG, RH, and VST.

### 2.4. Enzymatic Activity

The activity of endo-1,4-*β*-glucanase was measured using carboxymethylcellulose (CMC) as substrate as follows: 250 μL of 2% (*w*/*v*) carboxymethylcellulose in sodium acetate buffer 0.05 M, pH 4.8, was incubated with the same volume of the extracted sample at 50 °C for 30 min. Released glucose, the only reducing sugar liberated (previously confirmed by HPLC) from the CMC hydrolysis, was quantified by the DNS method. One unit of enzymatic activity was defined as the amount of enzyme needed to release 1 μmol/min of glucose under the conditions of the assay.

The procedure to determine xylanase activity was similar to the one described for endo-1,4-*β*-glucanase activity but using xylan 1% (*w*/*v*) as a substrate. Released xylose, the only reducing sugar released from xylan hydrolysis, was also quantified by the DNS method. One unit of enzymatic activity was defined as the amount of enzyme required to release 1 μmol/min of xylose at 50 °C and pH 4.8.

The activity of *β*-glucosidase was determined by incubating 100 μL of the substrate, 4 mM 4-nitrophenyl-*β*-D-glucopyranoside, in 50 mM sodium citrate buffer, pH 4.8, with 100 μL of the extracted sample at 50 °C for 15 min. To stop the reaction, 0.6 mL of sodium carbonate 1 M was added, following the addition of 1.7 mL distilled water. One unit of enzymatic activity was defined as the amount of enzyme needed to release 1 μmol/min of *p*-nitrophenol under the assay conditions.

Amylase activity was quantified using starch as a substrate. Briefly, 250 μL of 2% (*w*/*v*) starch substrate in sodium acetate buffer 0.05 M, pH 5.5, was incubated with 250 μL of the extracted sample at 40 °C for 30 min. The DNS method was used to quantify the released maltose. One unit of enzymatic activity was defined as the amount of enzyme required to release 1 μmol/min of maltose under the assay conditions.

Each enzymatic activity was expressed in units per gram of dry by-product (U/g).

### 2.5. Solid-State Fermentation

The SSF experiments were carried out in 250 mL Erlenmeyer flasks using 5 g of dry substrate. The initial moisture was adjusted to 75% (*w*/*w*) with distilled water. The flasks with substrates were sterilized at 121 °C for 15 min. Inoculation was conducted with 2 mL of spore solution of *A. niger* CECT 2088 with a concentration of 10^6^ spores/mL. Substrates were incubated for 7 days at 25 °C in the dark.

#### 2.5.1. Effect of Supplementation and Granulometry

The study of the effect of nitrogen and phosphorus supplementation on enzyme production was tested with RH and BSG as substrates. For this purpose, 2% (*w*/*w*) (NH_4_)_2_SO_4_ and 1% (*w*/*w*) K_2_HPO_4_ were added (4 mL of salt solution to 1 g of dry substrate), alone and in combination, to the substrate. A control fermentation without supplementation was also carried out.

The influence of particle size of the substrates was tested using three different granulometries: 10 mm, 4 mm, and 1 mm. These SSFs were performed with substrates supplemented with 2% (*w*/*w*) (NH_4_)_2_SO_4_ and 1%(*w*/*w*) K_2_HPO_4_. Each SSF was performed in duplicate.

#### 2.5.2. Mixture Optimization by Experimental Design

To determine the best mixture of by-products to be used as the substrate that maximizes enzyme activity, a simplex centroid mixture design was employed. In all the assays, inoculation, incubation parameters, and extractions were performed as described previously in Section 2.5. In total, nine assays were conducted with the three independent variables (BSG, RH, and VSTs) at different concentrations (*w*/*w*) (100%, 50%, and 33.3%) in a total amount of 5 g of dry matter. The dependent variables studied were the activity of endo-1,4-*β*-glucanase, xylanase, *β*-glucosidase, and amylase. Experimental data were evaluated by multiple regression analysis, resulting in the following equations (Equations (1) and (2)), which represent the linear and quadratic models, respectively:

*Y* = *β*_1_
*x*_1_ + *β*_2_
*x*_2_ + *β*_3_
*x*_3_ (1)

*Y* = *β*_1_
*x*_1_ + *β*_2_
*x*_2_ + *β*_3_
*x*_3_ + *β*_12_
*x*_1_
*x*_2_ + *β*_13_
*x*_1_
*x*_3_ + *β*_23_
*x*_2_
*x*_3_ (2)

where *Y* represents the predicted response variable (enzyme activity per mass of dry solid mixture, U/g), *β* represents the regression coefficients of the model, and *x* represents the independent variables (mass fraction of each by-product).

#### 2.5.3. Effect of Fermentation Time

Under the optimized SSF conditions that resulted from the experimental design for the overall enzyme production, fermentations were performed for 5, 7, 10, and 14 days. The activity of endo-1,4-*β*-glucanase, xylanase, *β*-glucosidase, and amylase was monitored in the SSF extracts, and SSF experiments were conducted using the same conditions mentioned above (75% moisture, 25 °C).

### 2.6. Statistical Analysis

All results are presented as the mean ± standard deviation (SD) of two replicates. Results were analyzed by one-way ANOVA, applying the Tukey multiple-comparisons test (*p* < 0.05). The experimental design was performed using Statgraphics Centurion XVI software (Manusgisties, Inc., Rockville, MD, USA).

## 3. Results

### 3.1. Effect of Phosphorous and Nitrogen Supplementation

**Figure 1:**
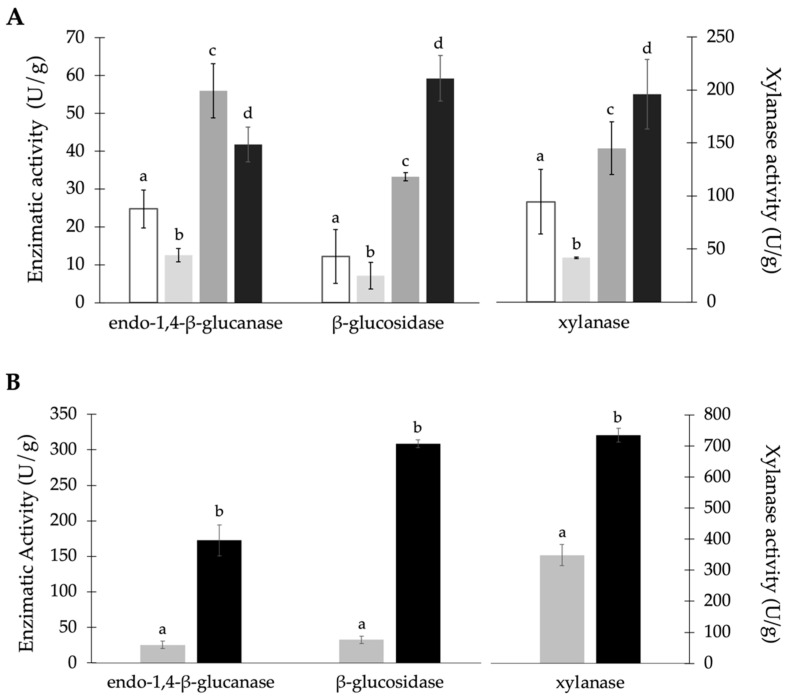
Activity of endo-1,4-*β*-glucanase, *β*-glucosidase, and xylanase in SSF by *A. niger* CECT 2088 in (**A**) RH and (**B**) BSG without supplementation (□) and with the supplementations of 1% (*w*/*w*) K_2_HPO_4_ (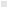), 2% (*w*/*w*) (NH_4_)_2_SO_4_ (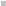), and both 1% (*w*/*w*) K_2_HPO_4_ and 2% (*w*/*w*) (NH_4_)_2_SO_4_ (■) after 7 days of incubation at 25 °C. Values with the same letter for the same enzyme and by-product do not present statistically significant differences (*p* ≥ 0.05).

Since RH was nutritionally the poorest by-product and had the lowest protein content of the three residues, it was used as the substrate to test the effect of nitrogen supplementation on SSF. SSF was performed with 2% (*w*/*w*) (NH_4_)_2_SO_4_, 1% (*w*/*w*) K_2_HPO_4_, and a combination of 2% (*w*/*w*) (NH_4_)_2_SO_4_ with 1% (*w*/*w*) K_2_HPO_4_. A control SSF was conducted without supplementation. Figure 1A shows the activity of endo-1,4-*β*-glucanase, *β*-glucosidase, and xylanase obtained after SSF.

In RH experiments, the maximum xylanase and *β*-glucosidase activities were obtained with the addition of both (NH_4_)_2_SO_4_ and K_2_HPO_4_ (Figure 1A). Xylanase activity increased about 2-fold, while *β*-glucosidase activity showed a 5-fold improvement in activity when compared with the control fermentation (without supplementation). The highest activity of endo-1,4-*β*-glucanase was obtained with only 2% (*w*/*w*) (NH_4_)_2_SO_4_ supplementation; however, with both supplementations, there was still an approximately 2.5-fold increase regarding the control assay. Therefore, the simultaneous supplementation of both components proved to be an effective approach for enhancing enzyme production using RH as a substrate.

This nitrogen and phosphorous supplementation was also tested using BSG as a solid matrix in SSF (Figure 1B). All the monitored enzymes were obtained at significantly increased activity levels. Xylanase, endo-1,4-*β*-glucanase, and *β*-glucosidase activities increased by 2-, 6-, and 8-fold, respectively, compared to non-supplemented BSG. Taking into consideration this positive impact of both supplements in BSG, which is the least recalcitrant by-product among the three, the addition of this component to the fermentation medium was kept in the following experiments.

### 3.2. Effect of Substrate Particle Size

**Figure 2:**
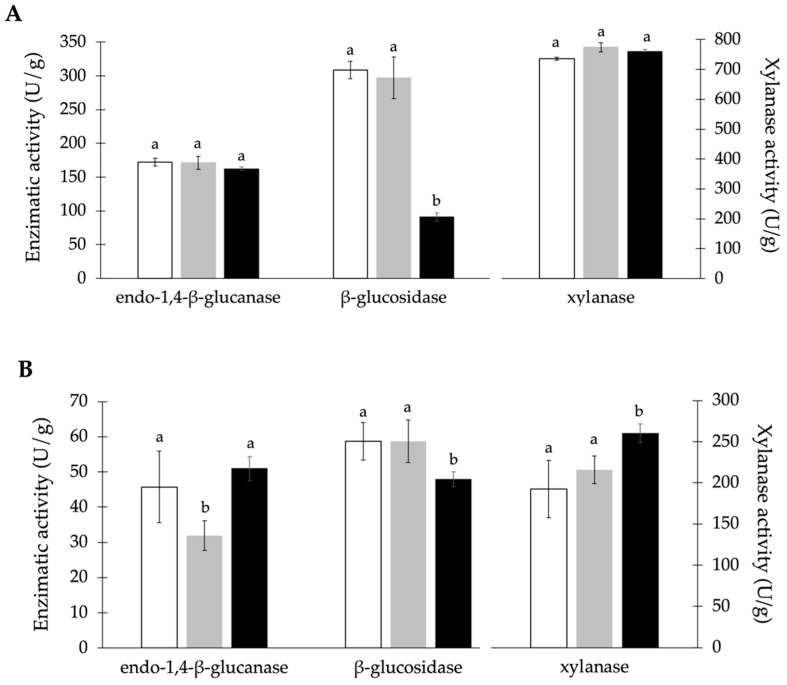

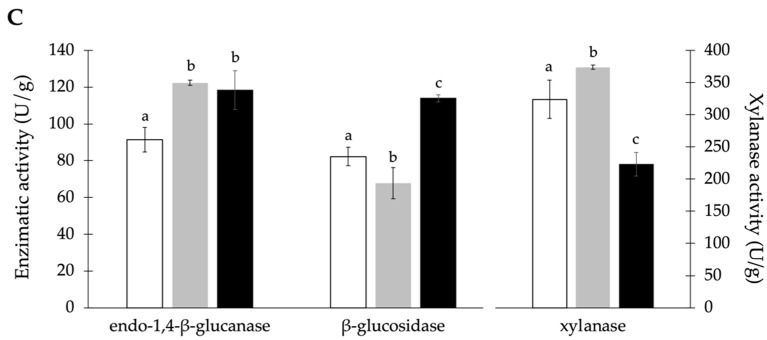
Activity of endo-1,4-*β*-glucanase, *β*-glucosidase, and xylanase by *A. niger* CECT 2088 at different particle sizes of (**A**) BSG, (**B**) RH, and (**C**) VSTs, i.e., 10 mm (□), 4 mm (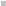) and 1 mm (■), during 7 days of incubation at 25 °C. All assays were supplemented with 1% (*w*/*w*) K_2_HPO_4_ and 2% (*w*/*w*) (NH_4_)_2_SO_4_. Values followed by the same letter for the same enzyme and by-product do not present statistically significant differences (*p* ≥ 0.05).

The dimensions of substrate particles play a critical role in microbial colonization, air penetration, and CO_2_ removal; consequently, fungal colonization, growth, and consequent produced enzymatic activity are dependent on the particle size of the solid used in SSF. Figure 2 shows the activity of endo-1,4-*β*-glucanase, *β*-glucosidase, and xylanase obtained in SSF with *A. niger* CECT 2088 at different particle sizes (1 mm, 4 mm, and 10 mm) of each by-product.

In the SSF of BSG, no significant differences in the activity of endo-1,4-*β*-glucanase and xylanase were observed as the particle size decreased. However, the smallest particle size (1 mm) resulted in a 70% decrease in the activity of *β*-glucosidase (Figure 2A). When using RH as substrate (Figure 2B), the use of a 1 mm particle size resulted in a 27% increase in xylanase activity, while 4 mm particles led to a decrease of 30% in endo-1,4-*β*-glucanase activity. Changes in particle size did not result in significant differences in the activity of *β*-glucosidase.

Enzyme production via the SSF of VSTs was affected by particle size variation (Figure 2C). The reduction in particle size from 10 mm to 4 mm increased xylanase and endo-1,4-*β*-glucanase activity by 15% and 25%, respectively, but *β*-glucosidase activity was slightly reduced. However, a particle size of 1 mm led to a 40% decrease in xylanase activity and a 38% increase in *β*-glucosidase activity when compared with the 10 mm substrate.

### 3.3. Optimization of Substrate Mixture

The optimal mixture of substrates for SSF to maximize enzyme activity was evaluated using a simplex centroid mixture design. Considering the results obtained in the supplementation and granulometry assays, all substrate mixtures were supplemented with 2% (*w*/*w*) (NH_4_)_2_SO_4_ and 1% (*w*/*w*) K_2_HPO_4_. In the SSF using BSG and RH as substrates, 10 mm particles were used, while for VSTs, a 4 mm particle size was selected. Amylase activity was also quantified in the following set of experiments. The composition of substrates for each SSF experiment and the enzymatic activity obtained are shown in Table 2. The use of BSG alone as the substrate led to the highest xylanase, amylase, and *β*-glucosidase activity, while the mixture of BSG and VSTs resulted in the best conditions for endo-1,4-*β*-glucanase production. The mixture of RH and VSTs with BSG had a positive impact on the production of all tested enzymes, with increased activities when compared with RH (4.6-, 5.2-, 5.7-, and 2.2-fold for xylanase, endo-1,4-*β*-glucanase, *β*-glucosidase, and amylase, respectively) and VSTs alone (1.6-, 1.2-, 2.3-, and 2.8-fold for xylanase, endo-1,4-*β*-glucanase, *β*-glucosidase, and amylase, respectively). The BSG and RH mixture produced only a slightly lower amount of xylanase and amylase (14% and 5% less, respectively) than BSG alone. Furthermore, the mixture of RH and VSTs improved the activity of xylanase and *β*-glucosidase compared with the use of only RH or VSTs.

The ternary mixture did not improve the activity of any of the enzymes when compared with the best condition, only leading to a higher enzymatic activity when compared with mixtures of BSG and VSTs, BSG and RH, and RH or VSTs alone for xylanase, *β*-glucosidase and amylase and with RH for endo-1,4-*β*-glucanase.

With this experimental design, it was possible to determine the optimal mixtures of the substrates that maximize enzymatic activity. The regression coefficients and statistical parameters of the simplex centroid mixture design are shown in Table 3.

For xylanase and endo-1,4-*β*-glucanase activity, a linear model was the best fit, while for amylase and *β*-glucosidase, a quadratic model was the most suitable. The statistical parameters showed a good fit of the models, confirmed by the R^2^ values (between 0.79 and 1.00). The models for all enzymatic activity are significant (*p* < 0.05), indicating the statistically significant relationship between enzymatic activity and the components of the mixture.

Each independent variable impact on enzymatic activity is directly proportional to the values of the regression coefficients. Positive coefficients represent synergistic interactions while negative coefficients indicate that the by-products in the mixture have an antagonistic effect. Apart from the coefficients *x*_1_*x*_2_ for xylanase activity and *x*_2_*x*_3_ for amylase activity, all the regression coefficients are positive, indicating a synergistic interaction for all substrates or mixture of substrates. Statistical significance was observed for every independent variable for endo-1,4-*β*-glucanase, xylanase, and *β*-glucosidase activity. For amylase, the mixture of substrates *x*_1_*x*_2_ and *x*_2_*x*_3_ did not have a significant effect on this enzyme’s production, with only *x*_1_*x*_3_ being statistically significant (*p* < 0.1).

**Figure 3:**
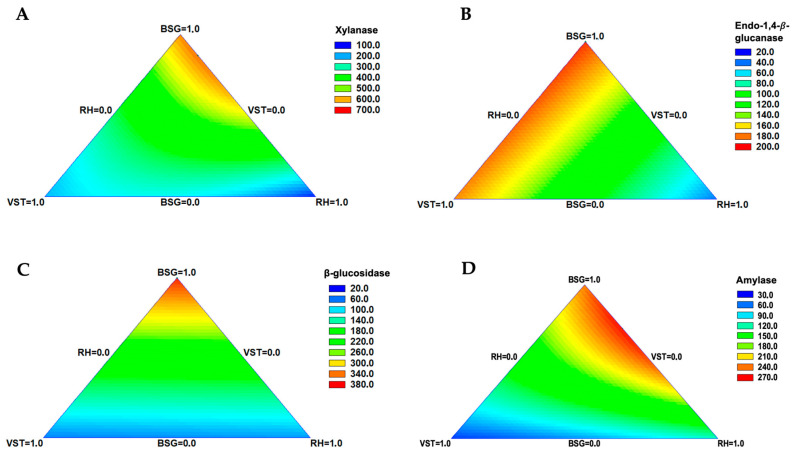
Contour plots for the dependent variables obtained in the simplex centroid mixture design for (**A**) xylanase, (**B**) endo-1,4-*β*-glucanase, (**C**) *β*-glucosidase, and (**D**) amylase. Values displayed in the color scales refer to enzymatic activity (U/g).

The variation in enzymatic activity using different amounts of the agro-industrial by-products is represented by the contour plots (Figure 3). In these plots, the vertex of each triangle corresponds to 100% of each one of the substrates in the study, with red regions representing the mixture of substrates where enzymatic activity is highest.

As shown in Figure 3, maximum xylanase and amylase activity was obtained by a binary mixture of BSG and RH, with red areas of the plot extending from the BSG to the RH vertex. *β*-glucosidase activity is higher when only BSG is used as a substrate, as can be seen by the red zone in the BSG vertex. The red regions in Figure 3B indicate that a binary combination of BSG with VSTs is the optimal substrate to maximize endo-1,4-*β*-glucanase production; however, regression coefficients obtained from the model indicate that BSG alone is the optimal substrate for this enzyme’s production.

The optimum values predicted by the fitted models of each enzyme’s activity are shown in Table 4.

The values obtained for response optimization indicate that, for endo-1,4-*β*-glucanase and *β*-glucosidase, single BSG is the optimal substrate that maximizes enzymatic activity, while xylanase and amylase activities are maximized by mixtures of BSG and RH. The predicted optimal values of enzymatic activity were also very similar to the ones obtained experimentally, particularly in the runs with BSG alone (Table 2).

### 3.4. Effect of Fermentation Time

**Figure 4:**
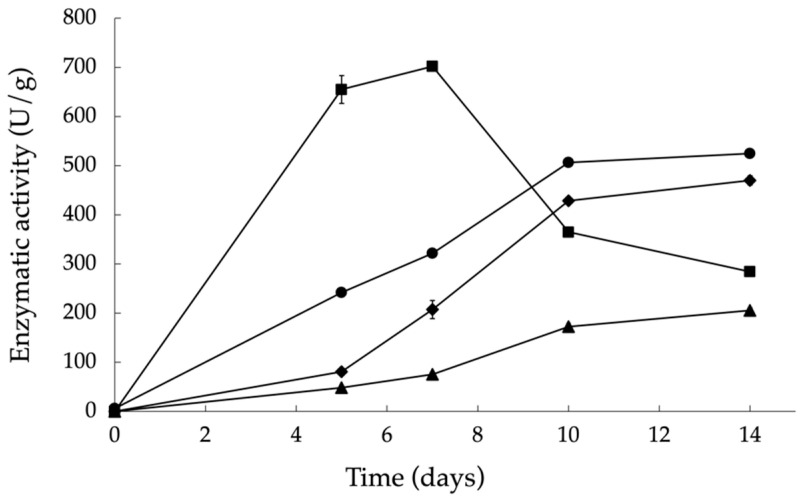
Enzymatic activity of endo-1,4-*β*-glucanase (▲), *β*-glucosidase (◆), xylanase (■), and amylase (●) over time in SSF of BSG supplemented with 1% (*w*/*w*) K_2_HPO_4_ + 2 % (*w*/*w*) (NH_4_)_2_SO_4_ using A. niger CECT 2088. The error bars represent the standard deviation of two independent experiments.

After the identification of BSG or the enriched BSG mixture as the overall optimal substrate that maximizes the production of the target enzymes, a study on different fermentation times was performed to evaluate the enzymatic activity produced at earlier or later times than the 7 days previously used (Figure 4). It was possible to conclude that for xylanase SSF can be shortened without reducing enzymatic activity (less than 7%), thus increasing xylanase productivity by 30%. After 7 days of SSF, which led to the maximum xylanase activity, the activity decreased significantly at the 10th and 14th days of fermentation. On the other hand, the activity of endo-1,4-*β*-glucanase, *β*-glucosidase, and amylase increased over time, with 3.6-, 5.3-, and 2.1-fold increases, respectively, between 5 and 10 days of fermentation, indicating that time is an important factor for selecting the enzymes to be produced.

## 4. Discussion

In recent years, there has been a notable increase in the lignocellulolytic enzymes market, driven by their extensive applications across various industries. It is estimated that the industrial enzyme market will reach USD 8.7 billion in 2026, growing at a compound annual rate of 6.3% (2021–2026 period), with lignocellulolytic enzymes accounting for more than 20% of the total revenue [34,35]. A further increase in the enzyme market is expected, driven by a growing demand in industries such as textile, leather, paper, and biodiesel, particularly in enzymatic biofuel production [36]. The commercialization of lignocellulolytic enzymes requires a sustainable production process that should, ideally, utilize low-cost substrates and minimize the use of chemicals, energy, and water while simultaneously achieving high yields and productivity and requiring reduced upstream and downstream processing. The by-products used in this work, namely brewer’s spent grain, rice husk, and vine shot trimmings, are thus potentially suitable as an economic substrate for lignocellulolytic enzyme production.

The physical–chemical composition of the solid substrates is of critical importance when regarding enzyme production. The content of cellulose and hemicellulose can promote the production of lignocellulolytic enzymes, such as cellulases and xylanases, respectively. Cellulases such as endo-glucanase, exo-glucanase, and *β*-glucosidase produced during SSF degrade the lignocellulosic matrix by attacking *β*-glycosidic bonds of the cellulose molecules, resulting in the production of monosaccharides (glucose). The target substrate of xylanases, xylan, is a major component of hemicellulose, which is converted into xylose by xylanase. While the production of these enzymes is induced in the presence of lignocellulosic material, it is repressed when easily accessible sugars are available for consumption [37].

The three agro-industrial by-products selected for this study had significant contents of lignocellulosic fibers (Table 1) and were successfully used to produce lignocellulolytic enzymes by SSF with the filamentous fungi *A. niger*. Considering the composition of these by-products, BSG has a greater potential to produce carbohydrases due to its higher protein and lower lignin contents when compared with RH and VSTs. Protein improves fungal growth during SSF, while lignin, a complex biopolymer, acts as a barrier to accessing cellulose and hemicellulose. Lignin biodegradation is a complex and highly energy-demanding process [38,39]. The values obtained for BSG’s cellulose and lignin contents were, respectively, higher and lower than those found in the literature [40]. These differences may result from the fact that BSG consists of a blend of several cereals with different compositions. Contrarily, RH had the lowest protein and highest lignin contents, which denotes its poor performance as a substrate for enzyme production by SSF. The high ash content of RH could also impair enzyme production [41].

Among the three by-products used in this work, BSG has been the most studied as a substrate for SSF with the goal of enzyme production by filamentous fungi [20,42,43,44]. For instance, Morán-Aguilar et al. [43] reported the use of BSG but studied the application of pre-treatments of the solid before SSF conducted with a higher content of water than the one used in this work. The maximum cellulase activity of 6.2 U/g reported by these authors was significantly lower than the values found herein, where no pre-treatments were applied. Liguori et al. [45] reported cellulase activity less than 50 U/g obtained in SSF by *A niger* LPB-334 of BSG supplemented with mineral salt solution and at a similar fermentation time used in this work, while for xylanase, activity values close to 1000 U/g were reported. Despite being a more recalcitrant by-product than BSG, some studies on the SSF of VSTs have been reported [42,46]. Vine shoot trimmings were previously fermented by *A. niger* and *A. ibericus* strains without supplementation [42], but the values reported of cellulases and xylanases activities were more than 6-fold lower than those obtained in this work. These values are also higher than the ones reported by Filipe et al., which obtained the activity values of 43 U/g, 30 U/g, and 10 U/g for xylanase, endo-1,4-*β*-glucanase, and *β*-glucosidase, respectively, in SSF with *A. niger* CECT 2915 [46].

Rice husk is an underexplored by-product, with few reports available on its use in SSF. da Silva Menezes et al. [47] reported activities of 118 U/g, 3 U/g, and 8 U/g for xylanase, endo-1,4-*β*-glucanase, and *β*-glucosidase, respectively, with fermentation using *Aspergillus* sp. These activities are considerably lower than those obtained in the current work (2-, 13-, and 8-fold lower for xylanase, endo-1,4-*β*-glucanase, and *β*-glucosidase, respectively). However, the supplementation, fermentation time, and temperature used by both authors were different.

The lack of nitrogen in some by-products has been reported as the main cause of the low microbial growth in these substrates. Thus, nitrogen and other elements supplementation has been reported as essential for effective SSF. This was also observed in this work, where the supplementation of the substrates with 1% (*w*/*w*) K_2_HPO_4_ and 2% (*w*/*w*) (NH_4_)_2_SO_4_) had a clear beneficial effect on enzyme production, resulting in a significant increase in the activity of all tested enzymes (Figure 1), even for BSG, which is a more nutritionally rich substrate than VSTs and RH. Gautam et al. [48] tested different nitrogen sources and concluded that the addition of 1% (*w*/*v*) of peptone to municipal solid waste residues led to the maximal production of cellulase using *A. niger*. Similarly, Liu et al. [49] concluded that in SSF with *Aspergillus fumigatus* Z5, maximal cellulase activity was achieved when peptone (1% *w*/*v*) was added to wheat straw. Lui et al. [50] showed that xylanase activity was positively affected by the supplementation of a substrate mixture of apple pomace and cottonseed meal with different nitrogen sources in SSF with *A. niger* SL-05. The optimal medium composition, obtained by response surface modeling, demonstrated that 2.5% (*w*/*w*) urea and 0.09% (*w*/*w*) KH_2_PO_4_ were the best supplementations, obtaining a xylanase activity of 4998 U/g. Oberoi et al. [51] reported that the addition of 2% (*w*/*w*) urea to the substrate (rice straw and wheat bran in a 4:1 ratio (*w*/*w*)) increased cellulase activity by 20% and that an increase of 50% in *β*-glucosidase activity was observed with (NH_4_)_2_SO_4_ supplementation in SSF with *A. niger* HN-2. In the current work, the addition of (NH_4_)_2_SO_4_ alone to RH led to a 2-fold increase in the activity of xylanase and endo-1,4-*β*-glucanase and a 3-fold improvement in *β*-glucosidase activity.

In this study, supplementation with K_2_HPO_4_, a source of phosphorous but also of potassium, had a negative effect on the activity of the three lignocellulolytic enzymes when compared with the non-supplemented control (Figure 1). Salihu et al. [52] showed that the addition of 0.04% K_2_HPO_4_ to soybean hulls led to the maximal production of this enzyme by *A. niger* AS-1. However, the authors observed a decrease in xylanase activity with high K_2_HPO_4_ concentrations. Also, Rodríguez [53] observed that 0.03% (*w*/*v*) and 0.02% (*w*/*v*) of KH_2_PO_4_ improved cellulase production by *Irpex lacteus* and *Pycnoporus sanguineus*, respectively. Thus, the decrease in the enzymatic activity observed in the experiments supplemented with K_2_HPO_4_ can be explained by the high concentration used (1% *w*/*w*) and by the fact that these elements were not the limiting factors of fungal growth and enzyme production, taking into account their contents in the by-products. Nevertheless, when nitrogen was supplemented and the fungi growth was not limited, further supplementation with K_2_HPO_4_ had a synergistic positive effect on enzyme production.

Lignocellulolytic enzyme production under SSF is also influenced by the particle size of the substrate. In this work, the particle size of the substrate had a significant effect on the production of endo-1,4-*β*-glucanase, xylanase, and *β*-glucosidase. The type of substrate also impacts the influence particle size has, with particle size variation in BSG only affecting *β*-glucosidase production, while for VSTs, all enzymes’ activities were affected by variations in the substrate size (Figure 2). Lower enzymatic activity obtained using smaller particle sizes may be attributed to an agglomeration of the substrate, leading to decreased porosity and low oxygen diffusion in the substrate matrix, which interferes with fungal growth [54]. However, it has been reported by several authors that smaller particle sizes lead to greater interaction between fungi and substrate due to the high surface area, which in turn can enhance enzyme production [55]. The literature usually reports SSF using smaller particle sizes (<5 mm) than those employed in this study [55,56,57,58], so comparisons of the influence of larger particle sizes (10 mm) in enzyme production are difficult to make. In this study, 10 mm, the original size of RH, was the best overall condition for SSF with BSG and RH, and thus further grinding of the solid to reduce its size is not justified. Studies with particle sizes close to the range used in this work obtained maximal enzyme activities with the following particle sizes: ≤ 5 mm using VSTs with *Aspergillus terreus* CECT 2808 [58]; 1 mm using RH with *Aspergillus brasiliensis* BLf1 and *Aspergillus nidulans* XynC A773 [57]; 2.2 mm using alkali pre-treated orange peels with *Emericella variecolor* NS3 [55]; 0.5 mm to 1 mm using microwave alkali pre-treated rice straw with *Aspergillus flavus* ITCC 6562 [59]; and 2 mm–3 mm using sorghum straw supplemented with glucose with *Thermomyces lanuginosus* D2W3 [56].

As discussed above, besides the structure and particle size, the composition of the substrate significantly affected the growth of fungi during SSF. The use of a sole agro-industrial by-product as a substrate can be insufficient to provide the essential nutrients required for fungal growth and enzyme production [60]. Therefore, the use of a mixture of various substrates can be a viable approach to enhance enzyme production by SSF. The use of simplex centroid mixture design allowed to find the optimal by-products mixture to improve enzyme production by SSF with *A. niger* CECT 2088.

Despite the best enzyme activities being generally obtained in SSF of BSG alone, endo-1,4-*β*-glucanase activity was slightly improved by mixing BSG with VSTs (Table 2, Figure 3). VSTs proved to be an effective alternative substrate for endo-1,4-*β*-glucanase production, alone or in combination with BSG, leading to interesting activity values. As predicted by the models, a combination of BSG with RH led to a maximum xylanase and amylase activity (Table 4 and Figure 3), while BSG alone produces the best activity values for endo-1,4-*β*-glucanase and *β*-glucosidase. BSG is a more widely re-used by-product and, due to its high protein content, has several applications in valued industries [61], while RH is a by-product of a highly recalcitrant nature that is mainly used to produce rice husk ashes by combustion. However, a vast quantity of the global production of rice husk and rice husk ashes remains unused waste materials [62,63]. The predicted synergic effect of combining RH with BSG in the production of two highly valuable enzymes (xylanase and amylase) can be a strategy to utilize a less valuable by-product such as RH while obtaining improved enzyme production.

Other studies evaluated the mixture of substrates to improve lignocellulolytic enzyme production. Filipe et al. [46] studied mixtures of olive pomace and winery waste to improve endo-1,4-*β*-glucanase and xylanase activity. Multiple response optimization demonstrated that xylanase, endo-1,4-*β*-glucanase, and *β*-glucosidase production were maximized with mixtures of 23% crude olive pomace (COP), 30% exhausted grape marc, 33% VSTs, and 14% exhausted olive pomace (EOP) for SSF with *A. niger* CECT 2915. Sousa et al. [23] reported that oil cake mixtures improved the production of xylanase, endo-1,4-*β*-glucanase, *β*-glucosidase, and protease compared to oilseed cakes alone. In SSF with *A. ibericus,* Sousa [64] concluded that a mixture of 54% BSG and 46% VSTs optimized the production of xylanase, endo-1,4-*β*-glucanase, and *β*-glucosidase, with optimal activities of 89 U/g, 73 U/g, and 22 U/g, respectively. Leite et al. [20] observed that a mixture of 55% BSG, 24% VSTs, and 21% EOP maximized endo-1,4-*β*-glucanase production (60 U/g) by *A. niger* CECT 2088, while 26% COP, 44% BSG, and 30% EOP led to a maximal xylanase production (791 U/g). The maximal *β*-glucosidase activity (273 U/g) was obtained by mixing 33% COP, 49% BSG, and 17% VSTs. Compared with these values, the current study obtained higher model-predicted activities for endo-1,4-*β*-glucanase and *β*-glucosidase with BSG alone (Table 4), while for xylanase the values obtained with the BSG and RH mixture were slightly lower.

As it was concluded that BSG was, overall, the best substrate for enzyme production, SSF was performed for different lengths of time from 5 to 14 days. It was verified that xylanase activity at 5 days is similar to that obtained at 7 days of SSF, decreasing abruptly at the 10th and 14th days of SSF. Other authors have reported that the maximum xylanase activity is achieved after 4 days to 7 days of fermentation, after which their activity starts to decline. For instance, Shata et al. [65] obtained the maximum xylanase production by *A. niger* NRC 9A after 4 days to 6 days of SSF. Also, Moran-Aguilar et al. [43] reported the highest xylanase activity after 5 days using *A. niger* CECT 2700 in SSF with BSG. These results also agree with those reported by Pérez-Rodríguez et al. [66] using the same *A. niger* strain with corn cobs as the substrate. The short 5-day incubation period can provide advantages such as lowering the risk of contamination and reducing costs associated with enzyme production [43]. Statistical analysis (orthogonal Taguchi design) performed by Morán-Aguilar and co-workers, indicated that the factor that most contributes to the expression of xylanases is fermentation time [67]. Prolonged incubation times lead to a sharp decrease in enzymatic activity, mainly due to the production of non-specific proteases secreted by the fungus, which could lead to the denaturation of synthesized xylanase.

The variation in the activities of endo-1,4-*β*-glucanase, amylase, and *β* -glucosidase over time was similar, with the three enzymes significantly increasing their activities from the 7th to the 10th and 14th days, with similar values for these two incubation times. Leite et al. [68] observed that the maximal endo-1,4-*β*-glucanase activity (35 U/g) was obtained after 11 days of SSF. Similarly, Casas-Godoy et al. [69] and Liguori et al. [45] verified that cellulases production by *Aspergillus fumigatus* and *A. niger* LPB-334 from BSG reached its peak at 11 days and 10 days, respectively. Suganthi et al. [70] reported that maximal amylase production by *A. niger* BAN3E using groundnut oil cake was reached after 6 days. Salgado et al. [71] showed that cellulase activity reached its peak after 6 days of fermentation with *Aspergillus uvarum* MUM 08.01, while *β*-glucosidase activity was higher after 8 days. The results found in the literature indicate that xylanase production starts earlier in the fermentation process, with cellulases being produced later [68,71]. This could be attributed to the need for the prior action of xylanases to expose cellulose fibers, thereby inducing cellulase production [72].

## 5. Conclusions

This study demonstrated that SSF with *A. niger* CECT 2088 is a suitable biotechnological process to produce lignocellulosic enzymes from brewer’s spent grain (BSG), rice husk (RH), and vine shoot trimmings (VSTs). BSG was the agro-industrial by-product that generated the highest production of lignocellulosic enzymes. Supplementation of the substrates with a nitrogen and phosphorous source significantly increased the enzymatic activity. The particle size of the substrate influenced enzymatic activity, with 10 mm being the overall best condition for enzyme production in BSG and RH, and 4 mm being the best for VSTs.

SSF was optimized using a simplex centroid mixture design that allowed the selection of 100% BSG as the substrate mixture yielding the optimal enzymatic activity of endo-1,4-*β*-glucanase and *β*-glucosidase, while a mixture of 87% BSG and 13% RH and 72% BSG and 28% RH were optimal for xylanase and amylase, respectively. Optimal activities of 189 U/g for endo-1,4-*β*-glucanase, 363 U/g for *β*-glucosidase, 627 U/g for xylanase, and 263 U/g for amylase were obtained. Despite these optimal mixtures, other mixtures including VSTs and RH, that is, less explored by-products, were proven to be feasible to obtain the target enzymes by SSF.

Xylanases were produced earlier on in SSF, reaching their highest activity values after 5 days of fermentation, while the other enzymes attained their highest activities on the 10th day, thus indicating that fermentation time can be a crucial operation factor in SSF contributing to target enzymes’ selectivity. The current study highlighted the potential of BSG, RH, and VSTs for lignocellulosic enzyme production, thus leading to the reduction in these by-products’ environmental impacts and contributing to a circular economy.

## Figures and Tables

**Table 1:** Composition of brewer’s spent grain (BSG), rice husk (RH), and vine shoot trimmings (VSTs) (% *w*/*w* in dry basis). Experimental values are the mean ± standard deviation of two analyses. Values with the same letter in each row do not present statistically significant differences (*p* ≥ 0.05).

**Parameter (%)**	**BSG**	**RH**	**VST**
Ashes	2.6 ± 0.2 ^a^	12.6 ± 0.3 ^b^	2.7 ± 0.1 ^a^
Protein	15.4 ± 1.2 ^a^	2.5 ± 0.1 ^b^	3.4 ± 0.1 ^c^
Total lipids	5.3 ± 0.6 ^a^	0.4 ± 0.3 ^b^	0.3 ± 0.1 ^b^
Klason lignin	6.6 ± 3.6 ^a^	25.1 ± 6.8 ^b^	21.0 ± 0.2 ^b^
Cellulose	40.9 ± 1.1 ^a^	37.1 ± 0.1 ^a^	39.7 ± 0.1 ^a^
Hemicellulose	14.9 ± 0.4 ^a^	19.8 ± 1.3 ^b^	22.7 ± 0.1 ^b^
**Minerals (g/Kg)**	**BSG**	**RH**	**VST**
Barium (Ba)	0.01	0.01	0.04
Calcium (Ca)	0.62	2.69	7.77
Copper (Cu)	0.01	0.01	0.03
Iron (Fe)	0.10	0.08	0.06
Potassium (K)	1.80	0.96	3.02
Magnesium (Mg)	0.57	2.18	0.84
Manganese (Mn)	0.08	0.04	0.04
Sodium (Na)	1.52	1.02	0.18
Phosphorus (P)	0.68	6.63	0.97
Strontium (Sr)	0.00	0.01	0.02
Zinc (Zn)	0.11	0.08	0.03

**Table 2:** Substrate composition for each SSF experiment of the simplex centroid mixture design and enzymatic activity (U/g) obtained in each run.

	BY-PRODUCT (% W/W)			ENZYMATIC ACTIVITY (U/G)			
RUNS	BSG	VST	RH	Xylanase	Endo-1,4-*β*-Glucanase	*β*-Glucosidase	Amylase
1	100	0	0	616	174	368	243
2	0	100	0	206	167	65	48
3	0	0	100	116	28	39	107
4	50	50	0	333	204	148	134
5	50	0	50	533	145	221	231
6	0	50	50	247	123	77	48
7	33.3	33.3	33.3	405	159	193	171
8	33.3	33.3	33.3	380	96	191	171
9	33.3	33.3	33.3	396	117	189	189

**Table 3:** Statistical parameters of the simplex centroid mixture design.

Parameter	Regression Coefficients	Xylanase	Endo-1,4-*β*-Glucanase	*β*-Glucosidase	Amylase
BSG	*x*_1_	615.3	189.1	362.8	237.7
VSTs	*x*_2_	206.0	175.0	62.7	43.7
RH	*x*_3_	115.4	40.0	71.2	102.7
BSG·VST	*x*_1_ *x*_2_	−305.8 **	-	-	41.9
BSG·RH	*x*_1_ *x*_3_	677.4 ***	-	-	311.9 *
VSTs·RH	*x*_2_ *x*_3_	350.0 **	-	-	−32.1
	Model (SS)	19,5548.0	16,936.7	73,008.5	38,621.4
	Total error (SS)	353.9	4418.5	7305.6	1631.5
	R^2^	1.00	0.79	0.91	0.96
	*p*-value	0.0003	0.0089	0.0008	0.0267

*** *p* < 0.001; ** *p* < 0.01; * *p* < 0.1; SS: sum of squares.

**Table 4:** Optimum values for each enzyme obtained by fitting multiple response variables to the optimal substrate mixture (% *w*/*w* dry basis).

	Xylanase	Endo-1,4-*β*-Glucanase	*β*-Glucosidase	Amylase
Predicted value (U/g)	627	189	363	263
Optimal substrate	87% BSG + 13% RH	100% BSG	100% BSG	72% BSG + 28% RH

## Data Availability

The data presented in this study are available on request from the corresponding author (accurately indicating status).
